# Predictors of multimorbidity among the Kurdish population living in the Northwest of Iran

**DOI:** 10.1186/s12889-020-09214-2

**Published:** 2020-07-11

**Authors:** N. Aminisani, L. Rastgou, S. M. Shamshirgaran, P. Sarbakhsh, S. Ghaderi, M. Hyde

**Affiliations:** 1 Statistics and Epidemiology Department, Tabriz University of Medical Sciences, Tabriz, Iran; 2 Healthy Ageing Research Centre, Faculty of Health Sciences, Neyshabur University of Medical Sciences, Janbazan Blvd, Neyshabur, Razavi Khorasan 9329774448 Iran; 3 District Health Centre, Bukan, Iran; 4 Centre for Innovative Ageing, College of Human and Health Sciences, Swansea University, Swansea, Wales

**Keywords:** Multimorbidity, Prevalence, Ethnic groups, Diabetes mellitus, Chronic disease

## Abstract

**Background:**

There is limited information about the predictors of multimorbidity (MM) among ethnic minority older adults in Iran. This study aimed to examine the impact of sociodemographic and lifestyle factors on the prevalence of MM, among older Kurdish people living in the Northwest of Iran.

**Methods:**

The current study was conducted in Bukan city located in West Azarbaijan province in the Northwest of Iran. The data for this study was based on the enrolment phase of a longitudinal study on ageing among the Kurdish population aged 50–94 yearswhich was designed according to the elderly component of the PERSIAN Cohort in Iran. Stratified random sampling was used to select people aged 50 + from eight health centres in Bukan from Oct 2017 to Dec 2018. Those who accepted the invitation and completed the baseline questionnaire were included in this study (*N* = 1493; Response rate 75%). A range of different factors,including sociodemographic and lifestyle factors as well as self-reported chronic conditions, was obtained by trained interviewers. MM was defined as “coexistence of two or more chronic conditions in the same person”. We included a list of 36 diseases/conditions in this analysis. Descriptive analysis and logistic regression were performed to compare the prevalence of MM according to different factors.

**Results:**

Over a third of the participants (36.6%) had ≥2chronic conditionsand 15.7% had≥3 chronic conditions. Hypertension, diabetes, musculoskeletal conditions, fatty liver, and heart diseases were common chronic conditions. In a fully adjusted logistic regression model, older age (OR_adj_ = 1.92, 95% CI: 1.48–2.48), being female (OR_adj_ = 1.49, 95%CI: 1.14–1.94), living without aspouse (OR_adj_ = 1.81, 95%CI: 1.34–2.44), and inadequate consumption of fruit and vegetables (OR_adj_ = 1.33, 95%CI: 1.06–1.67) were associated with the higher prevalence of MM.

**Conclusion:**

This study found that the prevalence of MM is relatively high among older Kurdishadults. Sociodemographic differences in the prevalence of MM might be of interest tothe health care system,and the prevalence of common chronic conditions in this study may highlight the need forlifestyle modifications in this community.

## Background

Population ageing has ledto concerns about increased multimorbidity (MM) which is defined asthe “coexistence of two or more chronic diseases in the same individual” [1]. It affects both individuals and health care systems,making this a global health challenge [2, 3].

A range of studies have investigated the prevalence and predictors of MM in different countries and reported the variableprevalence of MM, ranging from 13.1 to 71.8% around the world [4]. Results of a systematic review of studies conducted in primary care settings published between 1961 and 2013 included a total of 70,057,611 patients in 12 countries reported a variable prevalence ranging from 12.9 to 95.1% [5]. This variation might be explained by the heterogeneity in the data collection methods and the operational definition of MM [4, 6]. MM has been reported to be associated with poor quality of life [6], increase health care costs and utilization [7], functional decline [8] and death [9].

In Iran the proportion of older adults is rapidly growing, it is predicted that by 2050 the percentage of the aged population will increase to 29.4% and be comparable with that of the United States [10].Non-communicable diseases (NCDs) area leading cause of morbidity and mortality in Iran [11, 12]. Despite the high prevalence of chronic disease burden in Iran, research about MM in Iran is scarce, and few studies reported the prevalence of multimorbidity in overall and especially in ethnic minorities and questions have remained regarding the ethnic disparities in accumulation of chronic disease. The results of two studies based on data from the Golestan cohort study; a large-scale cohort study in a high incidence area of oesophageal cancer in northern Iran mainly among the Turkmen ethnic group,reported a prevalence of MM of 19.4%. Moreover, the study showed that women were more likely to present with MM [13, 14].

The most common chronic diseases reported in Golestan studieswere gastroesophageal reflux disease, cardiovascular diseases, diabetes and chronic obstructive pulmonary disease. Apart age and sex, other factors associated with a higher prevalence of MM were ethnicity, low socioeconomic status, physical inactivity, overweight/obesity, former smoking, opium and alcohol use [13, 14]. Iran is a multi-ethnic country which includes Persians, Kurds, Lurs, Arabs, Baluchs, Turkmen and Turkic tribeswith different sociocultural values influencing their lifestyles. Kurds are ranked as the third-largest ethnic groups following Persians and Azari people compromising about 10% of the population. They live mostly in the Northwest and West of Iran, which borders Iraq and Turkey. There is little known about the health and wellbeing of older Kurdish adults, especially as no published data is available about the prevalence of MM among this population [15]. Due to the disproportionate distribution of risk factors as well as the difference in social and cultural values, the prevalence and impact of MM might be different. This study could provide an insight into this problem and could provide important implications for improving primary and secondary prevention efforts to reduce multimorbidity-related health impacts to better plan and deliver care among ethnic groups.

The current study sought to address this lack of evidence by investigating the association between a range of sociodemographic, lifestyle factors and the prevalence of the MM in the Kurdish population. Previous data regarding the prevalence of MM and its related factors are based on the Golestan cohort study, which includes a specific minority group; Turkmen people mainly reside in rural areas. The current analysis was conducted in Bukan, West Azerbaijan Province, in the Northwest of Iran with an estimated population of 194,846 in 2017. To the best of our knowledge, this is the first study of its kind in the world to look at the prevalence of MM amongst Kurds living in Iran.

## Methods

### Study population

Urban residents of Bukan are almost entirely from the Kurdish-speaking community. Kurdish people follow their sociocultural values and mostly belong to the Sunnibranch of Islam. The data for this study was based on the enrolment phase of the BASwhich was a brief assessment of people aged 50–94 years using a similar questionnaire and measurement as an elderlycomponent of the Prospective Epidemiological Research Studies in IraAn (PERSIAN). In brief, the PERSIAN Elderly Cohort is the first comprehensive longitudinal study on ageing among people aged 50–94 years in Iran aims to assess the different aspects of ageing, monitoring changes in health and wellbeing of older adults using a wide range of data collection including a comprehensive questionnaire on demographic, socioeconomic, lifestyle, physical and psychological aspects, clinical examination, as well as mobility assessment, (www.persiancohort.com). Consideringprevalence of 19.4% based on the results of Golestan study, 1502 or more were needed to have a confidence level of 95% that the real value is within ±2% of the measured/surveyed value. Stratifiedrandom sampling was used to select the study populationfrom eight health centres in Bukan from Oct 2017 to Dec 2018.There was a recently updated list of people aged 50 years and older in these health centres from which we selected the study population. The participants of this study were residents of Bukan who were 50–95 years old without dementia, major depression and or disability which could limit their ability to participate in the study. The study staff invited them through a phone call. An interview was scheduled for those who accepted to participate in health centres during official hours. In overall, they contacted 2000 persons, of whom 341 (57% men and 43% women) were not interested in taking part in the study. Of those who accepted 166 were not eligible for and were not included. Theoverall response rate was 75% (*N* = 1493).

The study outcome was thepercentage of the population who reported having MM (≥ 2 chronic diseases). Thiswas based on the study participant’s response to the question ‘Has a doctor ever told you that you have any of the following health problems? We included a list of 36 diseases/conditions in this analysis including gastrointestinal conditions (peptic ulcer, Chron’s disease, ulcerative colitis, fatty liver) heart diseases and hypertension, neurologic diseases (stroke/Transient Ischemic Attack (TIA), epilepsy, Parkinson, migraine, headache, Alzheimer/dementia, MS), musculoskeletal (arthritis, osteoporosis),endocrine conditions (diabetes mellitus, hypo/hyperthyroidism), respiratory diseases (Chronic Obstructive Pulmonary Diseases (COPD), asthma), cancer, and mental disorder (depression, anxiety), and psychological disorders. Because selected non-communicable diseases (NCDs) such as diabetes, hypertension are recorded for national prevention and control of NCDs programs we were able to validate some of the respondents’ self-reported medical conditions against their medical recordsby interviewers under the supervision of a General Practitioner. However, because we used a more comprehensive list of chronic conditions in the survey than are recorded in the medical records we were not able to do this for all conditions.

### Data collection and preparation

Information for this study was collectedvia an interviewer-administered questionnaire. The interviewer also measured BMI via anthropometric height and weight. In analyses, a range of demographic, socioeconomic, lifestyle and clinical factors was examined. Age provided as a continuous variable also was categorised as 50–59, 60–69 and 70 years and over. Marital status was classified into two groups: married/living with a partner and divorced/separated/single/widow. Socioeconomic status indicators included educational qualification categorised as illiterate, and literate. Self-reported incomeadequacy was classified into 3 groups: I don’t have a problem, it is enough for basic needs, orit is not enough for basic needs. Smoking behaviour was based on whether respondents to identify themselves as a regular smoker or not. The Physical Activity Scale for the Elderly (PASE) was used to estimate the level of physical activity. It has beenvalidated in previous studies in Iran [16]. The PASE is a brief and specific instrument which has been designed for older adults to estimate physical activity recalled throughoutone week [17]. Frequency and time spent in avariety of activities including leisure time activities (walking; light activities, moderate, or strenuous intensity and muscle-conditioning activities) as well aswork-related activities (in paid or volunteer work) and household activities such as light house-work, yard work, and caring for others were also recorded. After considering the weight for each activity, the final PASE score for the week was calculated based on the sum of all activities, and the mean score was presented. While there are no specific cut points to categorize activity levels, our data were separated into tertile to classify physical activity levels as high (≥121), medium (56.5–120.9) or low (< 56.5) within each group for descriptive purposes; however the continuous score was used in regression analysis

Body Mass Index (BMI, kg/m2) was categorised as normal weight (< 25), overweight (25–29.9), obese (≥30) based on WHO-defined standard cut off points [12].Self-rated health status was categorized as excellent/very good/good, fair/poor. Adequate consumption of fruit and vegetables; was measured by asking questions about the number of raw and cooked vegetableserving/day as well as fresh fruit and juice, then a composite variable was made to classify the number of veg& fruit consumption per day. It was subsequently categorised into two categories: inadequate less than 5-A-day; adequate 5-A-day.

### Statistical methods

Differences in the characteristics of people with and without MMwere determined with the use of Student’s t-test for continuous variables and the chi-square test for categorical variables. We used the logistic regression modelling and calculated the odds ratios (ORs) and 95% confidence intervals (CIs) for multimorbidity by sociodemographic and lifestyle factors, and built three models; crude, age and sex-adjusted, and adjusted for socioeconomic and lifestyle factors (age, sex, education, marital status, income, BMI, physical activity, smoking, adequate fruit and vegetable intake, self-rated health status). Data were analysed using the STATA statistical package Version14, all estimates were reported with 95% confidence interval and a significance level 0.05.

## Results

A total of 1493participants responded to the baseline questionnaireand agreed to be followed up for the further waves of the study. The mean age of participants was 61.6 ± 9.5, a majority (70%) were less than 65 years of age. Of whom 62% were women, 82.5% were married and living with their spouses, 65.6% were illiterate, (only 6% had an education level of diploma and higher), only 21.7% reported that they have no financial difficulties and only38.5% were involved in in-paid work. The majority of participants were overweight/obese (79%), about 11.7% reported that they were current smokers, 58% reported inadequate consumption of fruit and vegetables (less than five serving per day), 25.5% reported their general health as fair/poor. The meanscore of PASE was 93.3 ± 61.7 ranged from 0 to 361.

Table 1 shows the baseline characteristics of the study participants who were with or without chronic conditions. The overall prevalence of multimorbidity (≥2 chronic conditions) was 36.6, and 15.8% (≥3 chronicconditions). As it can be seen relative to those without any chronic conditions, participants with two or moreconditions were older (*P* value< 0.001), women (*P* < 0.001),single/divorced/widowed (*P* < 0.001), had more income difficulties (*P* = 0.001) and had a higher frequency of overweight/obesity (*P* = 0.086). Compared to those without any chronic conditions, participants with MM reported more frequently their health as fair/poor (*P* < 0.001) (Table 1). Due to the disproportionate distribution of the men and women in our study furtherly, we calculated the weighted prevalence of MM in men was 39.9% and in women was 61.1%. The difference between unweighted and weighted results was not significant.

**Table 1:** General Characteristics of older Kurdish people living in the Northwest of Iran, the Bukan Ageing Study (BAS)

Variable	Number (%) total	Prevalence of < 2 chronic conditions, (%) rows	Prevalence of ≥ 2 chronic diseases, % row	***P*** value	Prevalence of ≥ 3 chronic diseases, % row	***P*** value
**Age group (mean + − SD)**	61.6 ± 9.5	60.3 ± 8.8	63.8 ± 10.3	< 0.001	65.8 ± 10.6	< 0.001
50–59	771 (51.6)	69.8	30.2	< 0.001	10.5	< 0.001
60–69	419 (28.1)	63.2	36.8		16.0	
≥ 70	303 (20.3)	49.8	52.5		28.7	
**Sex**						
Men	567 (38.0)	70.4	29.6	< 0.001	11.1	< 0.001
Women	926 (62.0)	59.2	40.8		18.6	
**Marital status**						
Married*	1232 (82.5)	67.0	33.0	< 0.001	13.7	< 0.001
Single/divorce/widow	262 (17.5)	46.6	53.4		25.6	
**Education**						
Illiterate	980 (65.6)	61.5	38.5	0.035	17.8	0.005
Literate	513 (34.4)	67.1	32.9		12.1	
**Family income**						
With major difficulties	172 (11.5)	51.7	48.3	0.001	22.7	0.029
Just enough	994 (66.7)	66.0	34.0		14.8	
No difficulties	324 (21.7)	61.4	38.6		15.4	
**Job status**						
In-paid work	575 (38.5)	71.3	28.7	< 0.001	10.1	< 0.001
Retired/no job	221 (14.8)	59.3	40.7		20.8	
Housewife	698 (46.7)	58.2	41.8		18.9	
**BMI**						
Less than 25	311 (20.9)	67.5	32.5	0.102	15.4	0.210
25–29.9	556 (37.3)	60.2	39.8		18.0	
≥ 30	624 (41.9)	63.9	36.1		14.1	
**Smoking (current)**						
*Yes*	175 (11.7)	58.3	41.7	0.133	18.9	0.228
*No*	1319 (88.3)	64.1	35.9		15.4	
**Fruit and vegetables**						
< 5 serving a day	846 (58.1)	59.6	40.4	< 0.001	17.6	0.053
≥ 5 serving a day	611 (41.9)	68.6	31.4		13.8	
**Self-rated health**						
Excellent/very good/good	1113 (74.6)	68.5	31.5	< 0.001	12.0	< 0.001
Fair/poor	380 (24.5)	48.7	51.3		27.1	
**Physical activity (Mean ± SD)**	93.3 ± 61.6	91.2 ± 61.3	96.9 ± 62.2	0.078	92.1 ± 61.9	0.768
Low (< 56.5)	511 (34.2)	66.1	33.9	0.216	17.4	0.374
Medium (56.5–120.9)	485 (32.5)	62.9	37.1		14.0	
High (> = 121)	498 (33.3)	61.0	39.0		15.9	

The results of a logistic regression adjusted for age and sex indicate that age, marital status, smoking, inadequate consumption of fruit and vegetables, and physical activity were associated with the prevalence of MM (≥2 chronic diseases) **(**Table 2**).** In a fully adjusted model, older age (Odds Ratio_adj_ = 1.92, 95% Confidence Interval: 1.48–2.48), being female (OR_adj_ = 1.49, 95%CI: 1.14–1.94), living without aspouse (OR_adj_ = 1.81, 95%CI: 1.34–2.44), and inadequate consumption of fruit and vegetables (OR_adj_ = 1.33, 95%CI: 1.10–1.67) were associated with a higher prevalence of MM. In a fully adjusted model including MM ≥3 chronic diseases as a dependent variable, only age (OR_adj_ = 2.30, 95%CI: 1.66–3.18) and sex (OR_adj_ = 1.90, 95%CI: 1.32–2.72) remained significant (data not shown).

**Table 2:** Predictors of multimorbidity among older Kurdish people living in the Northwest of Iran, the Bukan Ageing Study (BAS)

Variable	OR_**1**_^**a**^ (CI 95%)	OR_**2**_^**b**^ (CI 95%)	OR_**3**_^**c**^ (CI 95%)
**Age (Ref group 50–64)**			
≥ 65	2.12 (1.69–2.66)	2.16 (1.72–2.71)	1.92 (1.48–2.48)
*P* value	< 0.001	< 0.001	< 0.001
**Sex (Ref Men)**			
Women	1.63 (1.30–2.03)	1.67 (1.34–2.10)	1.49 (1.14–1.94)
*P* value	< 0.001	< 0.001	0.003
**Marital status (Ref Married/living with spouse**			
Single/divorce/widow	2.32 (1.78–3.05)	1.79 (1.27–2.27)	1.81 (1.34–2.44)
*P* value	< 0.001	< 0.001	< 0.001
**Education (Ref Illiterate)**			
Literate	0.79 (0.63--.98)	1.02 (0.80–1.29)	0.98 (0.76–1.26)
*P* value	0.035	0.895	0.885
**Income (Ref with major difficulties)**			
Just enough / No difficulties	0.58 (0.42–0.80)	0.75 (0.54–1.05)	0.86 (0.61–1.22)
*P* value	0.001	0.094	0.402
**BMI**^**d**^**(Ref less than 25)**			
≥ 25	1.26 (0.97–1.65)	1.15 (0.88–1.62)	1.18 (0.89–1.57)
*P* value	0.084	0.311	0.257
**Smoking (current) (Ref No)**			
Yes	1.28 (0.93–1.76)	1.44 (1.01–2.05)	1.40 (0.97–2.01)
*P* value	0.137	0.044	0.074
**Fruit and vegetable (Ref ≥ 5serving a day)**			
< 5 serving a day	1.48 (1.19–1.84)	1.33 (1.06–1.66)	1.33 (1.06–1.67)
*P* value	< 0.001	0.014	0.015
**Physical activity**			
Continuous variable	1.002 (1.00–1.003)	1.002 (1.00–1.004)	1.00 (1.00–1.004)
*P* value	0.082	0.019	0.034

^a^OR_1_: Crude Odds Rati ^b^OR_2_: Age &sex adjusted Odds Ratio ^c^OR_3_: Fully adjusted Odds Ratio (age, sex, education, marital status, income, BMI, physical activity, smoking, adequate fruit and vegetable intake, self-rated health status)

^d^*BMI* Body Mass Index (Weight (kg)/Height^2^ (m)

We reported the prevalence of certain chronic diseases to identify multimorbidity in this study population. We found that hypertension (31.5%), diabetes (17.3%), fatty liver (14.2%) and musculoskeletal conditions (21%) were the four chronic conditions most commonly reported. We found that women had higher rates of the majority of chronic diseases,while men had higher rates of cancer and stroke. We also found that except thyroid conditions, older adults(≥65 years old) had higher rates of classified chronic diseases (Table 3).

**Table 3:** Prevalence of common chronic conditions according to gender and age among older Kurdish people living in the Northwest of Iran, the Bukan Ageing Study (BAS)

Chronic disease	Prevalence (%)				
	Total	Men, %	Women, %	Age < 65, %	Age > =65, %
Hypertension	31.5	28.4	33.4	27.2	41.3
Diabetes	17.3	16.0	18.1	16.8	18.5
Heart Attack	1.9	1.9	1.9	1.5	2.9
Other heart conditions ^a^	9.5	10.2	9.1	8.6	11.5
Fatty Liver	14.2	10.7	16.3	13.2	16.6
Musculoskeletal conditions ^c^	21.0	13.9	25.3	17.1	29.6
Depression/ anxiety	6.5	4.1	8.0	4.7	10.6
Stroke	1.3	1.4	1.3	0.6	3.1
Respiratory conditions^b^	2.8	2.1	3.2	2.6	3.3
Hypo/Hyperthyroidism	3.6	1.8	4.6	4.3	1.8
Cancer (any type)	3.2	4.8	2.3	2.7	4.4
Kidney failure	0.8	0.4	1.1	0.6	1.3
Neurological conditions^d^	6.1	4.9	6.8	4.4	9.9

^a^Including Angina, Heart failure, Abnormal heart rhythm

^b^Asthma, Chronic Obstructive Pulmonary Disease, Chronic bronchitis

^c^Arthritis/ osteoarthritis/ osteoporosis

^d^Epilepsy, Multiple Sclerosis, Parkinson, Migraine, Headache, Alzheimer

## Discussion

Iran is facing the ageing of the population and a high prevalence of NCDs, as amulti-ethnic country, to deliver better health care and implement prevention strategies, ethnic variation should be taken into account. Little known about the prevalence of MM and its related health impact,especially in minorities. The current study aimed to estimate the prevalence of multimorbidity and the factors associated with it amongolder Kurdish people in the Northwest of Iran. To the best of our knowledge, this is the first such study of its kind. The overall prevalence of multimorbidity (≥2 chronic diseases) was 36.6% (95%CI: 34.2–39.1), and the prevalence of ≥3 chronic diseases was 15.7% (95% CI:14–17.7) among people aged 50 and over. Previous studies reported the variable prevalence of MM, ranging from 13.1 to 71.8% around the world [4]. Our prevalence was higher than the Golestan cohort study, which reported a prevalence of 19.4% [11]. As mentioned previously, the variation in the prevalence of MM might be explained by the heterogeneity in the data collection methods and the operational definition of the MM [4, 6]. In the current study, the long list of chronic conditions was included than Golestan study. Other possible explanation might be differences between urban and rural lifestyle; ourstudy population were urban residents while the majority were from rural areas in Golestan study. Our study participants were older than Golestan cohort study; the mean age in our study was 61.6 ± 9.5. and in that study was 52.1 ± 9 years. We should also mention that the self-reported method of data ascertainment in both studies might be suffering from a degree of underestimation, however, we attempted to verify some common conditions through the health records assessment.

There was a significant association between age and multimorbidity, many studies also reported the same results [18, 19], but our study found that it was about 30% of participants aged 50–59 years reported ≥2 coexisting chronic conditions and 11% reported≥3 coexisting chronic conditions which merit attention. In this study, MM was higher among women than menwhoare in line with many studies [4]. However, some studies reported no gender differences in patients attending primary care [20–22]. This can be explained by the difference in the study population and the list of diseases included, for example in a study conducted in Switzerland;a list of 75 chronic conditions derived from the International Classification for Primary Care was used.

Overweight/obesity was not associated with MM in the current study, which is opposite to other studies [12, 23]. That might be because the majority (79%) of our participants were overweight/obese. Overweight/obesity has become a global concern in recent years and is associated with a range of non-communicable diseases such as cardiovascular, diabetes, certain types of cancer and respiratory disease [24].

In this study, hypertension, diabetes, fatty liver, musculoskeletal conditions, and heart diseases were prominent conditions. Globally, hypertension as the leading preventable cause of death affects more than 30% of the adult population [25],and diabetes is an emerging health problem with an estimated about half billionprevalent cases in 2017 which is on the rise throughout the world [26]. Some studies included hypertension in the list of diseases for classification of MM [20] and some not [19]. This is because there is no consistency in the operational definition of MM yet, but diabetes and arthritis have been in the list of common comorbid conditions in published research [13, 20, 21].

### Limitations

Despite its strengths of being the first report of MM based on a population-based study among Kurdish ethnicity, this study has some limitations; mainly because of the definition of the chronic conditions and its verification. A self-reported method to ascertain the list of doctor-diagnosed chronic conditions seems to underestimate the prevalence of many chronic conditions. Although we attempted to verify the self-reported chronic conditions with the medical records in health centres, a degree of missing information still exits due to undiagnosed conditions. Besides, the difference between the quality of medical records in health centres between developing and developed countries is also another consideration. Comparability between the results of MM in men and women might be used with caution due to the more willingness of women than men to participate in the study and due to the timeframe of the data collection which was during official hours. This might be difficult for some men to attend the health centre and we did not have facilities to extend the working hours or arrange a home visit for them. There might also be a degree of selection bias due to non-respondents (25%). Although this is the first study among Kurdish community living in the Northwest of Iran., these people don’t represent all Kurdish people living in Iran. Furtherresearch in other Kurdish cities can contribute to draw abetter conclusion for the prevalence of MM among theKurdish population in Iran.

## Conclusions

This study found that the prevalence of MM is relatively high among Kurdish older adults. Sociodemographic differences in the prevalence of MM might be of interest tothe health care system, and the prevalence of common chronic conditions in this study may highlight the need for lifestyle modification in this community. In addition, to increase the case ascertainment, it is better to integrate information from different data sources.

## Data Availability

The data or analysis generated during this study is available from the corresponding author upon request.

## Abbreviations

MM: Multimorbidity
NCDs: Non-communicable diseases
BAS: Bukan Aging Study
TIA: Transient Ischemic Attack
BMI: Body Mass Index
CI: Confidence Intervals
COPD: Chronic Ostructive Pulmonary Diseases
PASE: Physical Activity Scale for the Elderly
OR: Odds Ratio
ORadj: Adjusted Odds Ratio
ORcrude: Crude Odds Ratio
